# Baculovirus Molecular Evolution via Gene Turnover and Recurrent Positive Selection of Key Genes

**DOI:** 10.1128/JVI.01319-17

**Published:** 2017-10-27

**Authors:** Tom Hill, Robert L. Unckless

**Affiliations:** Department of Molecular Biosciences, University of Kansas, Lawrence, Kansas, USA; University of Texas Southwestern Medical Center

**Keywords:** arbovirus, host-parasite relationship, population genetics, viral evolution

## Abstract

Hosts and viruses are locked in an evolutionary arms race. Hosts are constantly evolving to suppress virulence and replication, while viruses, which are reliant on host machinery for survival and reproduction, develop counterstrategies to escape this immune defense. Viruses must also adapt to novel conditions while establishing themselves in a host species. Both processes provide strong selection for viral adaptation. Understanding adaptive evolution in insect viruses can help us to better understand adaptive evolution in general and is important due to the use of these viruses as biocontrol agents and for protecting ecologically or economically important species from outbreaks. Here we examine the molecular evolution of baculoviruses and nudiviruses, a group of insect-infecting viruses with key roles in biocontrol. We looked for signatures of selection between genomes of baculoviruses infecting a range of species and within a population of baculoviruses. Both analyses found only a few strong signatures of positive selection, primarily in replication- and transcription-associated genes and several structural protein genes. In both analyses, we detected a conserved complex of genes, including the helicase gene, showing consistently high levels of adaptive evolution, suggesting that they may be key in antagonistic coevolution to escape host suppression. These genes are integral to the baculovirus life cycle and may be good focal genes for developing baculoviruses as effective biocontrol agents or for targeting baculoviruses infecting ecologically relevant species. Recombination and complex genomes make evolution in these double-stranded DNA viruses more efficient than that in smaller RNA viruses with error-prone replication, as seen via signatures of selection in specific genes within a population of baculoviruses.

**IMPORTANCE** Most viral evolutionary studies focus on RNA viruses. While these viruses cause many human and animal diseases, such studies leave us with a lesser understanding of how DNA viruses adapt to hosts and how the host responds to these pathogens. In this paper, we focus on the evolution of baculoviruses, a group of insect-infecting DNA viruses, many of which have been used in biocontrol. We find that most of the genome is under purifying selection, with only a few key genes evolving adaptively. Our results provide a glimpse into how DNA viruses differ from RNA viruses in their evolutionary dynamics and identify genes that are key to DNA virus adaptation, improving our understanding of how this group of pathogens evolves.

## INTRODUCTION

Antagonistic coevolution (arms races) of parasites and their hosts is common throughout the tree of life (1, 2). In fact, in several systems, immune genes and the parasites they regulate evolve rapidly, consistent with molecular coevolution of host and parasite (1–8). This is especially true for RNA viruses and host immune genes that suppress viral activity (8–11). Though RNA viruses are relatively simple, they are useful models for understanding the evolution of parasites in response to their hosts (12–15) as well as that of hosts in response to their parasites (16). Insect models are particularly good study systems due to the numerous immune pathways shared with humans and the lack of a true adaptive immune system, allowing the innate immune system to be investigated more directly (17, 18). Compared to RNA viruses, DNA viruses are less well understood in an evolutionary context (19). DNA viruses have a lower mutation rate due to higher-fidelity replication and larger, more complex genomes (19–23). This warrants a thorough analysis of DNA virus molecular evolution to more fully understand how these viruses coevolve with their hosts.

**FIG 1:**
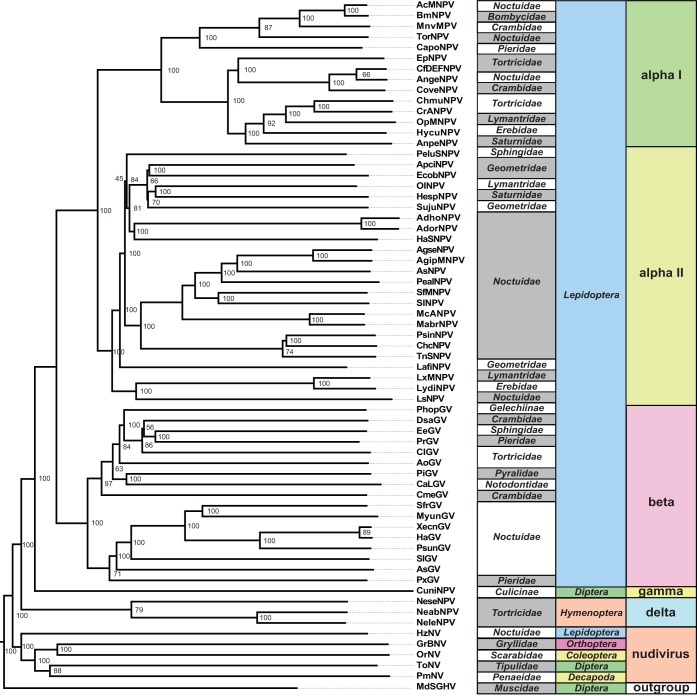
Phylogeny of viruses used for divergence analysis. The phylogeny is divided into alphabaculoviruses, betabaculoviruses, gammabaculoviruses, deltabaculoviruses, and nudiviruses. House fly salivary gland hypertrophy virus (MdSGHV) is included as an outgroup. See Data S1 in the supplemental material for genes found in all species, with full names and details. Node labels represent bootstrap support from the phylogeny assembled using PhyML (73). The figure also includes a table describing the order and family of the host of each virus.

Baculoviruses and nudiviruses are important DNA viruses because of their realized and potential use in biocontrol (24). For example, Oryctes rhinoceros nudivirus has been used as an effective method of biocontrol against Oryctes rhinoceros rhino beetles in Southeast Asia, reducing the extent of rhino beetle damage to local palms (24, 25). Baculoviruses are a group of rod-shaped viral particles with double-stranded DNA (dsDNA) genomes ranging in size from 80 to 180 kb (26). This diverse group of viruses is found in over 600 insect species (and also includes some arthropod-infecting nudiviruses), with various levels of virulence (27, 28). Baculoviruses can be divided into four main groups: the lepidopteran-infecting baculoviruses (alphabaculovirus groups I and II, subdivided due to differences in gene content), the lepidopteran-infecting betabaculoviruses, the dipteran-infecting gammabaculoviruses, and the hymenopteran-infecting deltabaculoviruses (Fig. 1). Nudiviruses are a closely related clade of viruses which infect a wide range of organisms, including organisms of the orders Diptera, Orthoptera, Coleoptera, Lepidoptera, and Decapoda (26–30) (Fig. 1). While alphabaculoviruses produce a protective protein matrix (the occlusion body) containing many virions for host infection, the remaining groups produce single virion capsules (nonoccluding viruses) (31). Interestingly, baculoviruses show little gene retention, with only a few core genes (involved in envelope production, replication, and RNA polymerases) conserved across the entire clade (32). Baculovirus genomes also show little synteny across the clade, likely due to their recombination-dependent method of replication, which reshuffles the gene order (26, 33).

Previous studies of the evolution of RNA virus populations within hosts found strong founder effects, population structure, and low diversity due to positive selection, all of which have helped to shape our understanding of (i) how viruses adapt to host systems, (ii) important genes for host infection, and (iii) potential complications of vaccination strategies (34–36). Despite our extensive knowledge of the species infected by baculoviruses and their routes of infection, we know little about baculovirus molecular evolution (22, 27). Because they are so different from RNA viruses and the more well-studied DNA viruses, it is difficult to predict which factors might influence selection or how the baculoviruses are evolving. With genome sizes often greater than 100,000 bp, on the order of 100 genes, relatively low mutation rates, and evidence of recombination allowing for more efficient selection (26, 37), baculovirus genomes may show signatures of natural selection unique among viruses. Recombination may also allow for the fine-scale identification of genes under selection. A deeper understanding of natural selection acting on baculoviruses in both the short term (within populations) and the long term (among different baculoviruses and nudiviruses) may elucidate important genetic components of the antagonistic relationship of host-virus coevolution. An understanding of these genetic components, such as identifying genes with variation that can aid in their escape from host suppression, will inform the use of baculoviruses in biocontrol and the protection of beneficial species, such as Bombyx mori (silkworms) or pollinators, from baculovirus outbreaks.

Here we explore the molecular evolution of baculoviruses to assess if these more genomically complex viruses undergo recurrent bouts of adaptation consistent with antagonistic coevolution with their hosts (as seen for RNA viruses) (14, 34, 36). We looked for signatures of selection across different baculovirus and nudivirus genomes and within a population of Autographa californica multiple nucleopolyhedrovirus (AcMNPV) to find genes consistently under positive selection (i.e., showing an increase in the frequency of advantageous genetic variants).

Primarily, we found strong purifying selection (the removal of variants that are deleterious) acting across the genome among nucleopolyhedroviruses; however, we found several genes associated with envelopes and capsids that are rapidly evolving within the population, as expected, to escape the host immune system. We also found that genes associated with viral replication and that form complexes with RNA polymerase are under recurrent positive selection, suggesting that these genes are common targets for suppression in the host.

## RESULTS

We collected whole-genome sequence data from 64 publicly available baculovirus and nudivirus annotated genomes (see Table S1 in the supplemental material). These sequenced genomes are from isolated cloned viral particles, which provided annotated, fully phased natural haplotypes (https://www.ncbi.nlm.nih.gov/genome/?term=txid10442[Organism:exp]). We determined gene orthology by reciprocal BLAST searches (38), resulting in 249 orthology groups and 20 open reading frames (ORFs) conserved among all baculoviruses and nudiviruses (Data S1). We used the concatenated amino acid sequence encoded by all conserved genes to create a maximum likelihood phylogeny by using PhyML. This phylogeny recovered the canonical viral clades (alpha-, beta-, gamma-, and deltabaculoviruses and nudiviruses) with high (79 to 100%) bootstrap support (Fig. 1).

**FIG 2:**
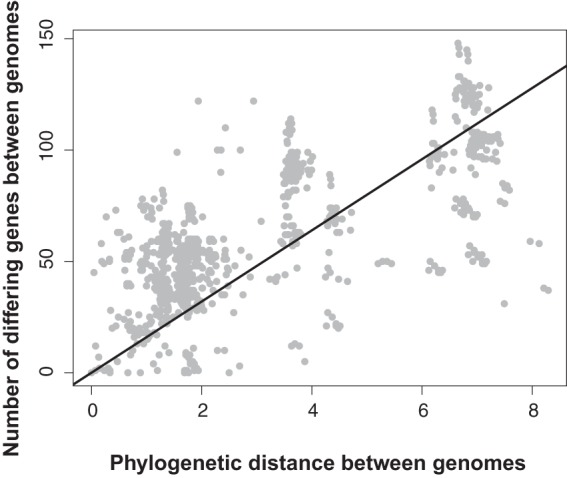
Divergence in gene content and sequence divergence of species pairs.

Previous work found various levels of gene conservation across baculoviruses, with increasingly different virus gene contents as viral genomes grow more divergent (26, 39). Consistent with this, we found a significant linear correlation between sequence divergence of viral genomes and divergence in gene content relative to that of a focal species for 59 of 64 focal species (for Spearman's rank correlation, *P* < 0.05 and ρ > 0.5; for Pearson's correlation, *P* < 0.05 and *R*^*2*^ > 0.5), with all comparisons showing a positive correlation between sequence divergence and gene content divergence (*R*^*2*^ > 0.15) (Fig. 2). Overall, the data show that distantly related viruses share fewer genes than those shared by more closely related viruses, as expected (Fig. 1 and 2; Data S1).

### Positively selected genes across the baculovirus phylogeny.

The availability of several annotated baculovirus genomes allows for inference of genes that have experienced positive selection across the baculovirus phylogeny. Genes experiencing positive natural selection show a higher ratio of nucleotide substitutions at replacement sites (nonsynonymous changes [*dN*]) to nucleotide substitutions at silent sites (synonymous changes [*dS*]), generally having an ω value or *dN*/*dS* ratio of >1 if the gene is under strong positive selection. We used two models in PAML (40) to estimate ω for each orthology group and to assess whether a model of positive natural selection fit the data better than a model of neutral evolution. We then localized this selection to codon sets by using the site test for selection; we also performed this analysis within specific clades to identify differences in positive selection between clades (40).

**FIG 3:**
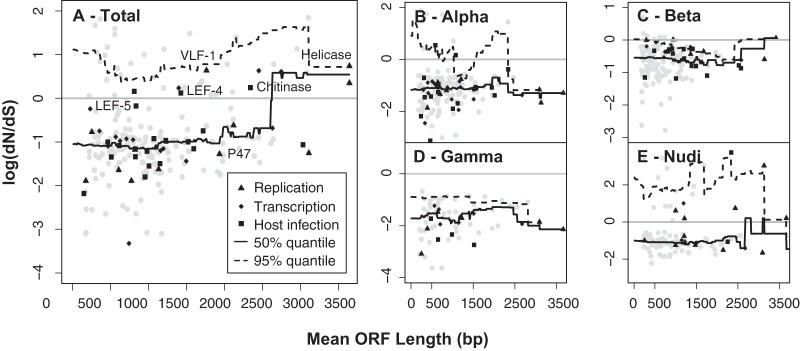
*dN*/*dS* values for genes by length for each model. *dN*/*dS* distributions for the M7 model across the whole tree and each viral clade are shown, and genes within the functional categories of replication, host infection, and RNA polymerase association are highlighted by different symbols. The 50th and 95th percentiles of *dN*/*dS* values were calculated using a 2,000-bp sliding window (sliding 200 bp). (A) Total; (B) alphabaculoviruses; (C) betabaculoviruses; (D) gammabaculoviruses; (E) nudiviruses.

Using trees and alignments generated by PRANK for each orthology group (41), we performed a PAML-based analysis of molecular evolution by using the site test of selection with models 0, 1a, and 2a (M0, M1a, and M2a; the site models) and models 7 and 8 (M7 and M8; the beta site models) (40). These models calculate the *dN* and *dS* for sites, with two (M1a and M7) acting nearly neutrally and two (M2a and M8) incorporating positive selection. These can then be used to identify selection occurring in codon sequences and to infer positions under positive selection by using two models (40). Using the PAML site-based models (M1a/M2a), we found M1a to be the best-fitting model consistent with neutrality. We could reject this model in favor of a model incorporating positive selection for 41 genes, and we were able to reject the M7 model in favor of the M8 beta site model for 42 of the 249 orthology groups. Overall, we found that 41 genes consistently had *dN*/*dS* values of >1 across both model types, supporting positive selection, including several genes of interest: *helicase*, *LEF-4*, *chitinase*, *IAP-2*, and *VLF-1* (Data S2). However, only one of these characterized genes (*helicase*) had a *dN*/*dS* value above the 95th percentile for gene length, highlighting its importance in the baculovirus life cycle (Fig. 3). We checked for gene tree discordance in the 20 conserved genes and all other genes with *dN*/*dS* values of >1 and found no drastic differences between the gene and genome trees; thus, we could not relate the adaptive evolution signatures to gene tree discordance (42).

Genes involved in viral transcription and replication had the largest proportion of ORFs under positive selection (>0.35 for both sets of tests in both groups), with similar numbers having *dN*/*dS* values of >1 for each functional group, suggesting that these categories are more likely to act antagonistically with the host system, resulting in this adaptation (Table 1). ORFs in both categories did not have significantly higher *dN*/*dS* values for fitting with a generalized linear model (GLM) (Table 2) and were not outliers in the permutation test (between the 52nd and 66th percentiles, based on 100,000 draws from the empirical distribution). *LEF-4* and *helicase* showed the strongest signatures of positive selection within these groups and were outliers in the *dN*/*dS* distribution (Fig. 3; Data S2). With the permutation test, we found that only hypothetical proteins were above the 95th percentile for both tests (Table 1). Additionally, the GLM showed no groups as significant outliers (Table 2).

**TABLE 1:** Summary of molecular evolution analysis^*a*^

Functional category	No. of genes	Site model (M0/M1a/M2a)				Beta site model (M7/M8)			
		No. of positively selected genes	Proportion of genes under positive selection	No. of genes with *dN*/*dS* value of >1	Median percentile	No. of positively selected genes	Proportion of genes under positive selection	No. of genes with *dN*/*dS* value of >1	Median percentile
Apoptosis	6	0	0	2	0.715	2	0.333	1	0.684
Capsid and envelope	20	4	0.2	3	0.539	6	0.286	2	0.644
DNA binding	11	2	0.18	2	0.624	2	0.18	2	0.714
Host infection	23	3	0.13	2	0.304	4	0.17	2	0.538
Hypothetical protein	158	21	0.13	29	0.571	17	0.107	30	0.464
Replication	14	5	0.35	4	0.524	5	0.35	4	0.670
Transcription	17	6	0.35	5	0.636	6	0.35	4	0.689
All	249	41	0.16	47		42	0.168	45	

a The data are separated by the site-based test (testing for selection at specific amino acids, using the M1a and M2a models) and the beta site test (testing for selection at specific sites, using a different set of parameters in the M7 and M8 models), implemented by PAML codeML. We considered genes to be under positive selection if the best-fitting model was M2a for the site-based models and M8 for the beta site models. The median percentile given is the probability that the median of the functional group is an outlier of the empirical distribution by chance.

**TABLE 2:** Generalized linear model for *dN*/*dS* versus functional group

Functional group or intercept	Model parameter		*t* value	*P* value
	Estimate	SE		
Intercept	1.008	0.4571	2.205	0.0284
DNA binding	−0.14755	0.07913	−1.865	0.0623
Envelope and capsid	−0.6825	0.5221	−1.307	0.1924
Host infection	−0.5455	0.5311	−1.027	0.3055
Hypothetical proteins	0.1536	0.4671	0.329	0.7426
Replication	0.2681	0.5598	0.475	0.6353
Transcription	−0.2613	0.5431	−0.481	0.6309

### Different genes show signatures of positive selection across individual clades.

Among genes inferred to be evolving under positive selection, we found several differences between clades (alpha-, beta-, and deltabaculoviruses and nudiviruses) (Data S5). In the alphabaculovirus clades, several proteins associated with diverse functional categories (host infection, envelope, and apoptosis) showed evidence of positive selection. Based on permutation tests, however, the only outliers for *dN*/*dS* values were hypothetical proteins (100,000 draws from the empirical distribution) (Table S2). By identifying specific genes above the 95th percentile based on gene length (using 1-kbp sliding windows), we found *LEF-7*, *LEF-10*, *FGF*, *ODV-E66*, *p35*, and *p43*. These genes are primarily involved in transcription and envelope formation.

Across the betabaculovirus clade, eight genes showed evidence of positive selection, with *dN*/*dS* values of >1, including six hypothetical protein genes, *helicase*, and *LEF-7*. We found that genes involved in viral replication were in the upper 95th percentile for *dN*/*dS* values (based on the permutation test, using 100,000 draws from the empirical distribution). Additionally, the following five genes were above the 95th percentile for *dN*/*dS* values based on length: *helicase*, *LEF-7*, *LEF-6*, *FGF*, and *enhancin* (encodes a metalloprotease thought to facilitate infection by degrading host membranes) (Fig. 3; Table S2).

In the deltabaculovirus clade, only three genes showed signatures of positive selection in the beta site model, including two hypothetical protein genes and *helicase*. Again, the only functional category with an excess of positively selected genes was the hypothetical protein category. Within the nudivirus clade, a larger proportion of genes showed signatures of positive selection than the case for other clades (0.18; <0.08 for all other clades). Most genes which showed signatures of positive selection (*dN*/*dS* values of >1) were associated with replication and RNA polymerase, with both categories in the upper 95th percentile (using 100,000 draws from the empirical distribution). Across the phylogeny, we found similar signatures of positive selection in several key genes, including *helicase*, *LEF-4*, *VLF-1*, and *AC92* (Fig. 3).

A hallmark of adaptive evolution across a phylogeny is evidence of multiple substitutions at the same codon. We identified significantly positively selected amino acid substitutions throughout the PAML results with *dN*/*dS* values of >1 and *P* values of <0.05 for more than one clade. Only 113 individual amino acid substitutions in 23 orthology groups showed consistent patterns of positive selection among clades. Of these substitutions, 39 were found in the envelope/capsid group, with 30 in one gene, *vp91*, suggesting that *vp91* may be a constant target of suppression by the viral host. Additionally, 10 amino acid substitutions in the DNA polymerase were found to be positively selected across the different clades, as well as 12 in IAP-2, an apoptosis-suppressing factor. Both may be factors which are adapting to the various host systems infected by these viruses. Across the nine genes with multiple shared substitutions, eight genes had selected codons all clustered within the gene; however, only *IAP-2* and the DNA polymerase gene had substitutions in any functionally annotated regions (26).

### Positively selected amino acids across the baculovirus phylogeny.

Across the entire phylogeny, we found significantly positively selected substitutions in 195 of the 249 orthology groups. By sorting these genes by functional category, we found a significant excess of positively selected sites in genes involved in capsid, host infection, transcription, and hypothetical proteins (logistic regression; *P* < 0.05) (Table 3). There was also a significant deficit of positively selected sites in replication, transcription, and envelope proteins (Table 3).

**TABLE 3:** Logistic regression analysis of the number of significantly selected codon sites in each functional group

Functional group or intercept	Model parameter		*z* value	*P* value
	Estimate	SE		
Intercept	−4.4236	0.1043	−42.406	<2e−16
DNA binding	0.1730	0.1258	1.375	0.169
Envelope and capsid	−1.2254	0.1400	−8.751	<2e−16
Host infection	0.1870	0.1126	1.661	0.0467
Hypothetical proteins	0.3512	0.1073	3.274	0.00106
Replication	−0.7675	0.1216	−6.310	2.80e−10
Transcription	0.8023	0.1351	0.821	0.244

A majority (112 genes) of the positively selected genes encode hypothetical proteins with no known function or functional domains, which precludes a finer-scale analysis of domains under selection. The *helicase* gene, encoding a key replication protein, is among these functional genes with identifiable adaptive substitutions (Data S4). Although most of the substitutions were found at the 5′ end of the *helicase* sequence, we were unable to localize these to any domain because no domain contained a higher *dN*/*dS* value than that for the rest of the sequence. In addition to *helicase*, many genes for late expression factors (LEFs), including *LEF-4* and *LEF-5* (Data S4), showed signatures of adaptation.

In *LEF-5*, 6 of the 9 selected substitutions are in the coding region for the C-terminal zinc-binding domain (26). *LEF-6*, encoding a transcription-associated protein, showed the largest number of positively selected amino acids for all characterized proteins, with an excess of sites under positive selection, though we found no evidence that these were localized to any specific functional domains (Data S4).

### Fast-evolving genes are found in fewer baculovirus genomes.

Given the high rate of evolution of some viral genes, orthology may be more difficult to assign for genes under positive selection. Consistent with this, 34% of orthology groups were found in only a subset of the genomes analyzed (Data S1 to S3). This may be because fast-evolving genes are more difficult to assign to orthology groups or because of genuine gain and loss across the clade. A large number of these gene groups have not been categorized properly (grouped as hypothetical protein genes) and thus may actually be orthologous to each other, but we are unable to group them (26, 39).

**FIG 4:**
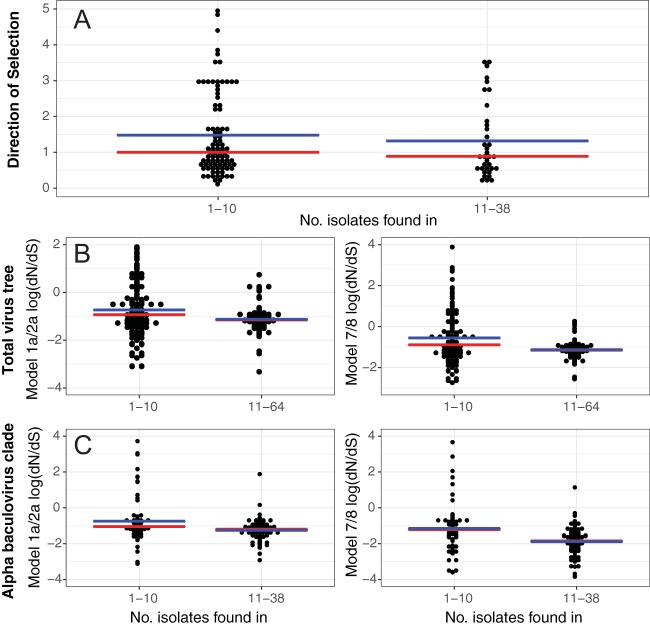
Measures of selection by number of genomes. (A) McDonald-Kreitman neutrality index (direction of selection). (B) PAML site model and beta site model across the total viral tree. (C) PAML site model and beta site model across the alphabaculovirus tree. Each plot is binned by number of species in which the genes are found. Bins are separated into 1 to 10 and 11 to 38 or 64 species, as 10 species appears to be the cutoff for higher *dN*/*dS* values (Data S2).

To test if these genes are evolving significantly faster than those found in a larger number of species, we grouped our *dN*/*dS* values based on the number of species in which each gene was present and found that the *dN*/*dS* value was significantly higher for genes found in fewer than 10 species in both the site-based model and the beta site model across the entire tree and the alpha clade (Wilcoxon rank sum test; M1 *W* = 8,055 [*P* < 0.007]; M7 *W* = 8,166 [*P* < 0.003]) (Fig. 4). Consistent with this, we found that >48% of significantly positively selected amino acid substitutions were in hypothetical proteins in orthology groups found in fewer than 10 genomes (Data S2 and S3). It is entirely possible that some outliers may have orthologous matches with hypothetical proteins that were missed by this survey due to their high rate of molecular evolution, which may explain the higher *dN*/*dS* values for several hypothetical proteins.

### Evidence of natural selection within a population of baculoviruses.

We moved from the assessment of molecular evolution of distantly related viruses to that of evolution within a viral population to identify possible candidate genes under positive selection. To this end, we analyzed whole viral genome sequences of Autographa californica nucleopolyhedrovirus (AcMNPV) from 20 naturally infected individuals found in a wild population of the host organism, Autographa californica (Table 4) (43). We used the individual-wise single nucleotide polymorphism (SNP) frequency to infer the population-wide frequency, based on SNP calls from Lofreq and GATK Haplotypecaller (44, 45). We also cross-referenced these called SNPs with a previous set of variants to make sure that no sequencing errors were included alongside true variants (43), excluding their effect on the identification of signatures of adaptation.

**TABLE 4:** AcMNPV sequences used for population-level analysis

Accession no.	Line ID	Read no.
SRR1119903	S0	10019526
SRR1120009	S1	14944099
SRR1120065	S2	36195764
SRR1120362	S3	25939880
SRR1120103	S4	2806575
SRR1120106	S5	10893999
SRR1120170	S6	7927299
SRR1120269	S7	6695187
SRR1124059	S8	10479029
SRR1124050	S9	10214302
SRR1119904	T0	10181720
SRR1119940	T1	7814487
SRR1120007	T2	6921780
SRR1120010	T3	6863315
SRR1120012	T4	7481923
SRR1120015	T5	8247633
SRR1120016	T6	23589731
SRR1120102	T7	17090438
SRR1120104	T8	13058831
SRR1120105	T9	6349924

Recent selection is inferred when a set of sequences have an excess of low-frequency derived alleles, indicating that a mutation recently swept to fixation and left little variation near the site (46). Tajima's D statistic allows for the detection of this: a negative Tajima's D value is consistent with a recent selective sweep at a gene due to an excess of low-frequency derived polymorphisms (47). We calculated Tajima's D across the genome in sliding windows and by gene, using called SNPs and their estimated frequencies in the total viral pool.

**FIG 5:**
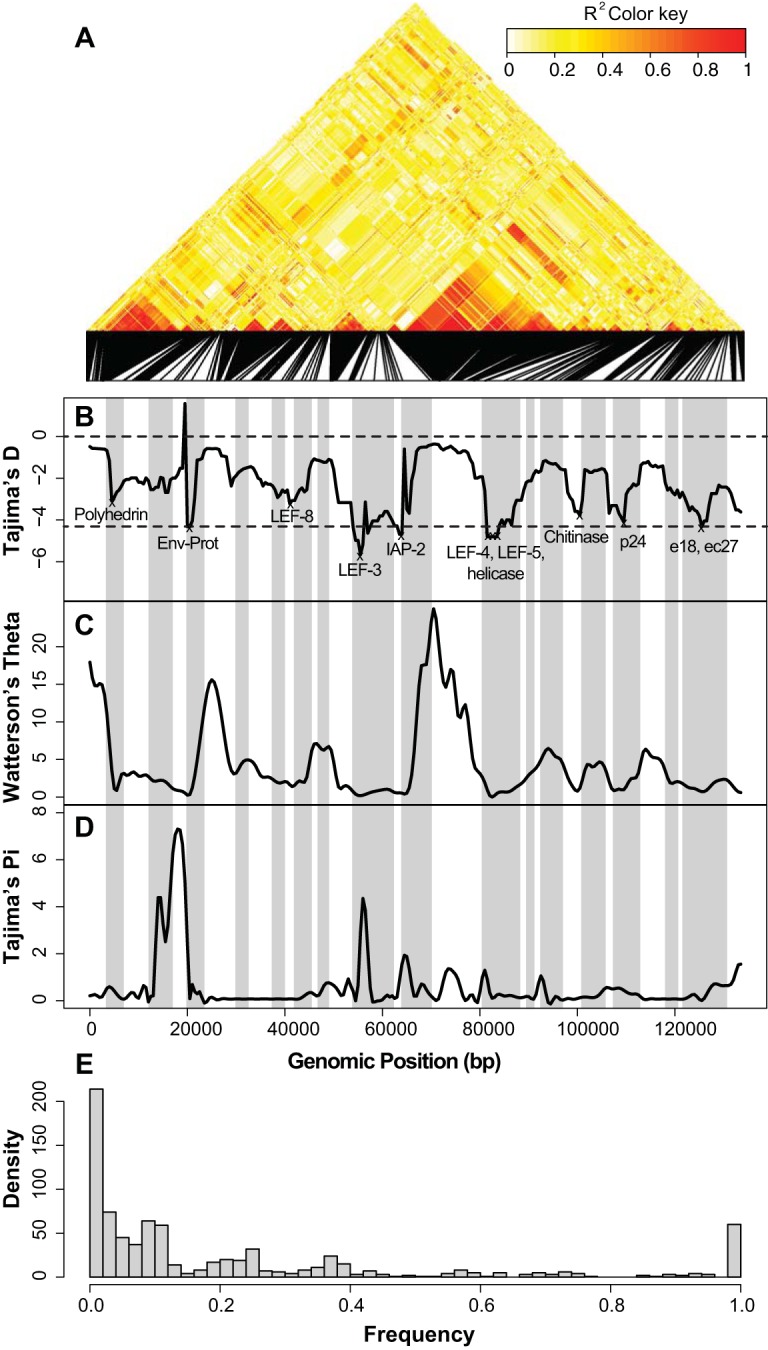
Summary statistics across the AcMNPV genome. (A) Linkage map of the AcMNPV genome showing linkage disequilibrium (*R*^2^ values). (B) Tajima's D distribution. Windows which are significantly lower than expected values have potential causal genes highlighted. The lower dashed line is positioned at the lower 5% quantile of Tajima's D, while the upper dashed line is at 0. The shaded regions are windows which have fewer than 2 detectable recombination events per 1 kbp (window size = 5 kbp; step size = 1 kbp). (C) Watterson's theta by sliding window (window size = 5 kbp; step size = 1 kbp). (D) Tajima's pi by sliding window (window size = 5 kbp; step size = 1 kbp). (E) Unfolded site frequency spectrum.

Tajima's D is consistently negative across the genome. This may be due to selection acting on the entire genome or, more likely, to a recent demographic history that involves changes in viral population size (as expected for infectious agents) (Fig. 5B). A consistently low Tajima's D value might be due to selection if recombination is low enough that neutral sites can hitchhike to fixation along with sites in selected genes (those involved in replication, host infection, and envelope production). Figure 5 shows that genes under selection tend to be in valleys of low Tajima's D values, consistent with a recent selective sweep in that region. We found nine genes below the 5% quantile of Tajima's D values (−4.432), eight of which are in the envelope production, replication, host infection, or transcription category, with one hypothetical protein gene. Again, *helicase*, *IAP-2*, and *Env-Prot* were found among the selected genes (Fig. 5B).

Previous work with RNA viruses identified founder effects caused by the bottlenecking of viral populations upon initial infections (34). These founder effects can lead to the fixation of neutral or deleterious polymorphisms due to their presence in the starting population. A population expansion following a bottleneck can produce the same signature as a selective sweep, possibly leading to the misidentification of positive selection. Though we did see several fixed private substitutions across samples (4% of polymorphic sites) and an excess of SNPs at 20% frequency in the site frequency spectrum (Fig. 5E), we found no difference in any population-level analyses (Tajima's D and direction of selection for each gene) if these sites were removed from the data (Welch two-sample *t* test; *t* < −0.69824; df = 270.41; *P* > 0.4856) and did not find any differences in genes below the 95th percentile; therefore, we did not remove these SNPs from the population-level analyses. We also calculated pairwise *F_ST_* (the fixation index, a measure of population differentiation) values between the virus pools in different hosts and found specific polymorphisms that showed high *F_ST_* values (*F_ST_* > 0.5) between at least one pair of hosts for SNPs in nine genes. Three of these genes encode hypothetical proteins, and the remaining genes are *p47*, *LEF-9*, *cg30*, *LEF-4*, *helicase*, and *chitinase*. While these high *F_ST_* values were found for only 39 of 171 pairwise comparisons, did not show up in global *F_ST_* surveys, and were not consistent for each gene in each case, it is worth highlighting that population structure may affect our results, as four of these genes showed signatures of selection in our population scans.

Recombination can aid in the inference of selection, as it can prevent a selective sweep from causing linkage disequilibrium between a sweeping locus and a linked neutral locus by breaking down the association of these two loci. A sweep can also remove variation from loci, giving the appearance of a lack of recombination in these regions (48). We estimated the recombination rate of AcMNPV across the genome by using the consensus viral sequence from each infected individual. Despite high levels of recombination across the ∼130-kbp genome, we found two main areas of strong linkage, each neighboring a region possibly under positive selection, based on low Tajima's D values (Fig. 5A and B). Consistent with a recent selective sweep, we also found few SNPs for detecting recombination events in regions with Tajima's D values below the 5% quantile and thus were unable to properly estimate recombination rates in these areas (Fig. 5B).

**FIG 6:**
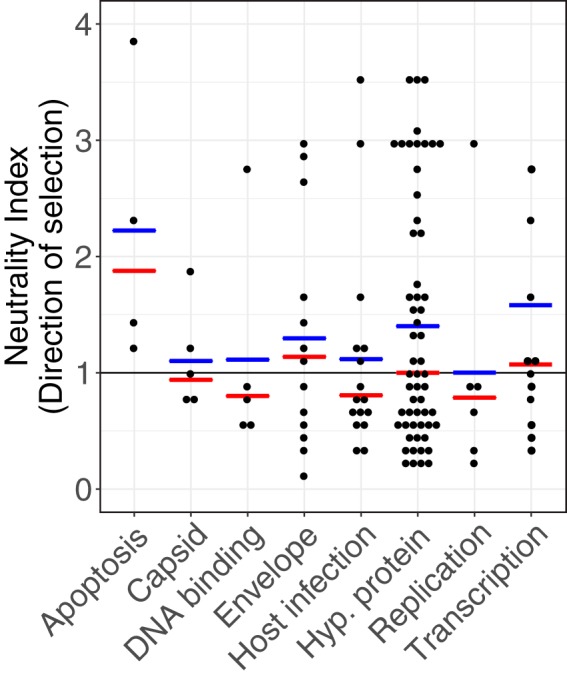
Distribution of the neutrality index, an estimate of the direction of selection of a gene, calculated using the McDonald-Kreitman test. Genes are binned by broad functional categories. The median of each category is shown in red and the mean in blue.

A negative Tajima's D value can identify candidate regions or genes that recently experienced a selective sweep but is unable to indicate whether those genes have been the subject of recurrent positive selection. Recurrent positive selection is inferred by use of polymorphism and divergence data, by comparing the ratio of replacement polymorphism to replacement divergence to the ratio of silent polymorphism to silent divergence, using the McDonald-Kreitman (MK) test (49). Using polymorphism data from AcMNPV and divergence data from Bombyx mori nucleopolyhedrovirus, we performed MK tests to calculate the neutrality index (also known as the direction of selection) and again binned genes by broad functional category (Fig. 6).

**FIG 7:**
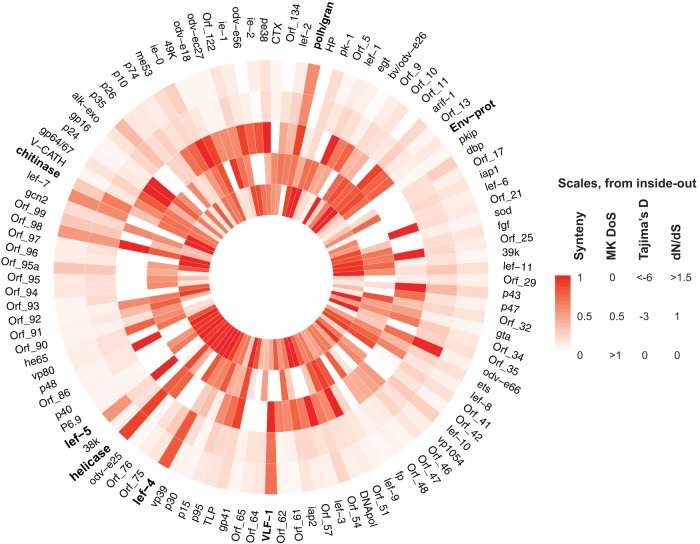
Map of the AcMNPV genome, showing associations between measures of selection. From the inside out, the bars show position conservation, alpha, Tajima's D, *dN*/*dS* from the site model, and *dN*/*dS* from the beta site model. Values taken are gene based. The gene order given is based on the position in the AcMNPV genome. DoS, direction of selection.

Four genes showed significant differences from neutrality in the MK test (Fisher's exact test [FET]; *P* < 0.05), suggesting positive selection (Table 5). Again, we found genes involved in replication and host infection showing strong evidence of positive selection. Additionally, based on the direction of selection, we found further evidence of positive selection in several other genes involved in host infection and envelope/capsid production, with 10 and 9 genes, respectively, under positive selection (index for direction of selection of <1). We found *helicase* among the selected genes (Table 5; Fig. 6). We also found genes for DNA polymerase, the envelope proteins EC27 and p30, the transcription factor 49K, ARIF-1 (an actin-associating factor necessary for host infection) (50), and 28 hypothetical proteins of unknown function. These results further support the idea that envelope proteins are evolving antagonistically with the host to escape host recognition and suppression. Further, sweeps on key replication and host infection proteins suggest that these may be undergoing similar “arms races” to escape suppression (Fig. 7).

**TABLE 5:** Summary of McDonald-Kreitman test results for a population of AcMNPV

Functional category	No. of genes	Neutrality index (direction of selection)			
		Mean	Median	SD	No. of genes with FET *P* value of <0.05^*a*^
Apoptosis	4	2.223	1.877	1.190	0
Capsid	6	1.051	0.870	0.435	0
DNA binding	4	1.249	0.823	1.037	0
Envelope	12	1.310	1.048	1.019	0
Host infection	16	1.334	0.861	1.086	1
Hypothetical protein	63	2.156	1.333	2.762	2
Replication	6	1.001	0.786	1.016	1
Transcription	14	1.509	0.833	1.616	0

a FET, Fisher's exact test.

## DISCUSSION

It stands to reason that most viruses, with their small number of genes, parasitic lifestyle, and often inherently error-prone nucleic acid replication process, are evolving quickly and that their genes show signatures of positive natural selection. In most studies of viral evolution, positive selection is rampant (e.g., for HIV [51], influenza virus [52, 53], West Nile virus [54], and cytomegalovirus [55]) (reviewed in reference 19).

Others have reported widespread purifying selection in some viruses, such as cotton leaf curl virus (56, 57). Most of this work has been completed for RNA viruses, yet there has been no comprehensive treatment of the molecular evolution of large double-stranded DNA viruses. We expect DNA and RNA viruses to show contrasting patterns of evolution due to differences in genome size, mutation rates, and methods of replication (19). Baculoviruses (and the closely related nudiviruses) are a large group of double-stranded DNA viruses that infect insects (28). Their large genomes, apparent recombination, and sequence availability make them ideal for studying DNA virus molecular evolution and suggest that they might evolve very differently from other, smaller viruses with more error-prone replication (26). Though we saw primarily evidence of purifying selection, we also saw evidence of strong positive selection (i) within a population of viral particles, (ii) within viral clades, and (iii) across baculovirus clades, consistent with antagonistic coevolution of virus and host organisms. However, we observed only moderate overlap in the classes of genes selected at each level (Tables 1 to 3, 5, and 6). Strong evidence of positive selection is found in very few genes, likely because recombination allows more efficient selection on smaller genomic regions in these viruses.

**TABLE 6:** AcMNPV genes with strong signatures of positive selection within and between genomes^*a*^

Gene	Category	Position in AcMNPV (kb)	Mean *dN*/*dS*	MK DoS^*b*^	Tajima's D	Positive selection across lineages	Positive selection in AcMNPV
*Polyhedrin*	Envelope	4.5	0.077	0.611	−5.463	No	Yes
*Env-Prot*	Envelope	18.5	0.238	0.575	−4.644	No	Yes
*LEF-8*	Transcription	40.5	0.269	0.430	−3.787	No	Yes
*LEF-3*	DNA binding	57.7	0.308	2.793	−8.171	No	Yes
*IAP-2*	Apoptosis	61	0.404	1.455	−4.684	No	Yes
*LEF-4*	Transcription	76.5	1.264	0.762	−1.528	Yes	Yes
*helicase*	Replication	80.6	2.092	0.223	−4.802	Yes	Yes
*chitinase*	Host infection	105.2	0.925	0.605	−1.907	Yes	Yes
*V-CATH*	Host infection	106.9	0.251	0.671	−3.59	No	Yes
*p24*	Capsid	109.9	0.229	1.182	−6.01	No	Yes
*e18*	Envelope	125.1	0.161	3	−3.52	No	Yes
*ec27*	Envelope	125.3	0.127	0.083	−3.615	No	Yes

a Positions and gene names are given based on the AcMNPV genome, with mean *dN*/*dS* values giving the means of the best-fitting beta site and site model values for the given genes' orthology groups.

b DoS, direction of selection.

**FIG 8:**
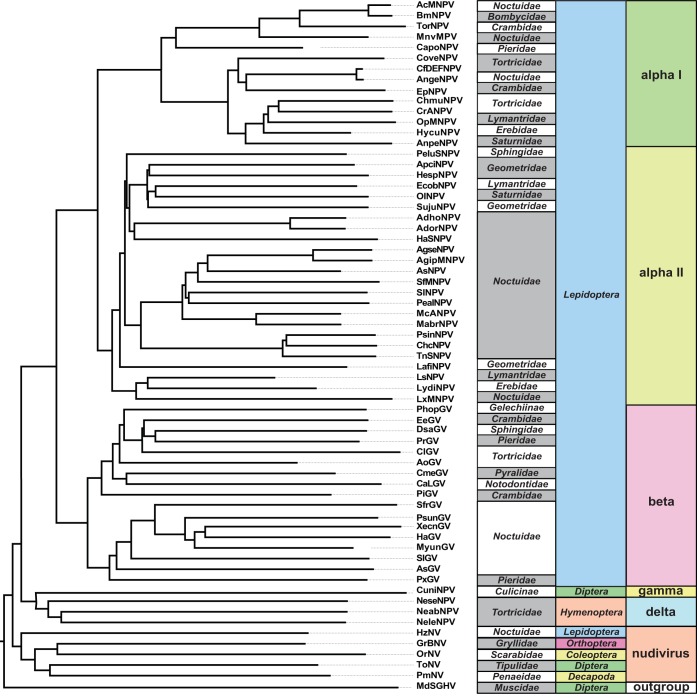
Phylogeny assembled by hierarchical clustering of species based on the presence of orthology groups found in this study.

The differences observed between the divergence- and polymorphism-based analyses may highlight the fact that a single viral genome (AcMNPV in this case) is adapting to pressures different from those for the entire baculovirus clade. In support of this, we found that each baculovirus clade shows a different set of genes under positive selection. We expect the different signatures of selection across clades to tie back to host specialization (Fig. 1 and 8). These viruses are constantly coadapting with their differing hosts, with different genes showing signatures of positive selection as a function of the exact host they infect. We therefore expected to find fewer genes consistently under positive selection in nudiviruses, with their diverse range of hosts. Fitting our expectations, only genes associated with replication and components of the RNA polymerase showed signatures of positive selection, like the genes under positive selection across the entire baculovirus phylogeny.

Within a population of AcMNPVs, we found a largely different set of genes under selection. While we still found positive selection in *helicase*, *LEF-4*, and *LEF-5*, we also found several positively selected genes involved in host infection and associated with the viral envelope and capsid. The primary candidates for adaptation here are *Env-Prot*, *polyhedrin*, and *ec27*. Most of these genes are involved in viral envelope formation and can act as antigens for viral recognition within the host. It might be expected that these genes contain complexes that evolve rapidly to avoid detection by the host system.

Possible explanations for the differing signatures of selection within and between populations are the invalidation of the infinite sites model due to recurrent mutations and the occurrence of frequent bottlenecks followed by a population expansion coupled with differences in recombination rate (which could lead to the various values for Tajima's D across the genome) (58). However, the overlapping signatures of positive selection found using Tajima's D and the McDonald-Kreitman-based approach (which should be robust despite these effects) (49, 59) instill confidence that these genes are in fact under positive selection (Fig. 7) (58).

Due to the high levels of divergence across the baculovirus phylogeny, saturation of synonymous substitutions may yield a false signature of positive selection. This would be evident if, with high levels of nonsynonymous divergence, synonymous divergence began to level off as it became saturated. We found a significant positive association between *dN* and *dS* (*R*^*2*^ = 0.2163; *P* = 1.883e−14), with all genes with *dN*/*dS* values above the 95th percentile having *dS* values in the lower 25th percentile. This suggests that saturation of synonymous sites did not influence our results.

As baculoviruses and nudiviruses diverge in sequence due to various selective pressures, they are also diverging in gene content, although some of this apparent divergence may be due to the fast-evolving group of hypothetical proteins, whose orthology is difficult to assign. It would be interesting to ascertain whether a phylogeny based on viral gene content is more congruent with host phylogeny than that based on the viral DNA sequence. Unfortunately, there are insufficient data to confidently estimate the host phylogeny with the available data, and published phylogenies based on molecular data for lepidopterans disagree with each other. This precludes a proper analysis of gene conservation among distantly related viruses with similar hosts. By clustering viral genomes by the absence or presence of orthology groups, we found congruency based on baculovirus groups (alpha I, alpha II, beta, gamma, delta, and nudivirus), though we did find some rearrangements within these groups, possibly due to the gain or convergent loss of genes between genomes. This difference between the viral genome phylogeny and the gene content phylogeny together with the few genes showing signatures of positive selection suggests that gene turnover is rapid and either that nonvital genes are frequently lost or that gene gain/loss is adaptive, with most substitutions occurring in few genes and virulence factors turning over rapidly.

Though it appears that some genes are frequently lost, several genes appear to be both present and positively selected across the phylogeny, suggesting not only that these genes are essential for baculovirus function but also that they may play a role in host infection and be in antagonistic coevolution with their host. We found that genes involved in replication show signatures of positive selection (Tables 1 to 3, 5, and 6), as do several RNA polymerase components. These factors may be constant targets of suppression by the hosts, resulting in their rapid adaptation in all baculoviruses. In addition to this, we found a complex of genes that not only are retained in all viral genomes but also are syntenic across all genomes and show linkage disequilibrium within a population (Fig. 5 and 7). The region from *p48* to *LEF-4* is retained in the same arrangement in each genome, as previously found for the region from *helicase* to *LEF-5* (32). Previous work found that this core set of genes is essential for baculovirus function and comprises one transcriptional unit and/or that their positional retention is vital for baculovirus replication (32).

**FIG 9:**
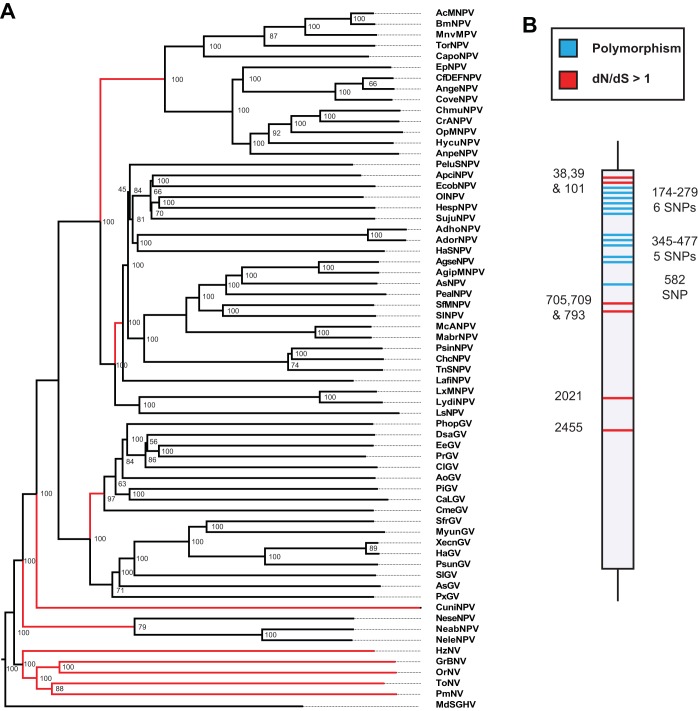
Regions of positive selection in *helicase*. (A) Phylogeny of baculoviruses, with branches of positive selection in *helicase* highlighted in red. (B) Sites of positive selection in the *helicase* sequence, as found using codeML, and polymorphic sites found in the population of AcMNPV.

The helicase gene appears to be the most consistent target of selection found in our survey and is an obvious target for the host immune system. Without a functioning copy of *helicase*, viral DNA is left compact and is unable to properly replicate. DNA helicases also have a conserved helicase domain, which makes their genes easily recognizable (26). In this case, we would expect specific key regions in the helicase domain to constantly be adapting to escape host suppression. Following this logic, we found that many positively selected sites occurred in the first half of the gene in both the population and divergence analyses (Fig. 9). However, differing from a constant host-parasite coevolutionary conflict, most positively selected substitutions in the divergence analyses occurred in branches leading to a change of host, suggesting that they may in fact be adaptations to a new host in some cases (Fig. 9).

Interestingly, *helicase* is a member of the syntenic cluster of genes all showing signs of positive selection. Alongside *helicase*, *LEF-4* and *LEF-5* are also found in this cluster and are known to be essential genes for baculovirus and nudivirus function (26). However, given the strong signatures of positive selection for these two genes across all surveys and the strong block of synteny containing these two genes, they may be more important for baculovirus replication within the host than previously recognized. *LEF-4* encodes a component of RNA polymerase and is involved in mRNA capping of viral transcripts, allowing them to be processed by host machinery. Similarly, *LEF-5* encodes an accessory factor for viral RNA polymerase and is necessary for the initiation of some aspects of transcription (26). Hosts may target *LEF-4* and *LEF-5* to prevent further transcription and the processing of viral mRNA. Since these factors, alongside *helicase*, not only are necessary for viral replication but also are maintained in a syntenic block in all baculoviruses and nudiviruses, they are ideal long-term targets for suppression of viral activity by the host, resulting in an antagonistic coevolution of hosts and parasites.

*IAP-2* and *chitinase* are also positively selected both within populations and between genomes (Fig. 7; Table 6). Both factors facilitate host infection, with *chitinase* likely being acquired from hosts before the alpha/beta divergence (60). In baculoviruses, chitinase acts to disintegrate the infected host cells upon cell death, facilitating the dispersal of viral particles within the host (61, 62). Baculoviruses without functional chitinases leave intact cells, reducing the dispersal rate as these cells are then degraded by the host (26, 61, 62). It is in the host's best interest to prevent the action of this chitinase, suggesting that it may be important for host-virus coevolution. *IAP-2* prevents the premature apoptosis of infected host cells, allowing for completion of the baculovirus replication phase. Again, a host that can suppress the action of *IAP-2* can reduce the viral load (26, 63), and this dynamic may lead to an “arms race” between host and virus. These genes that are known to be important in the antagonistic coevolution of host and pathogen may provide insight into biocontrol mechanisms.

Since most, if not all, alphabaculovirus chitinases contain predicted KDEL-like sequences, while betabaculovirus chitinases typically lack these (64), viruses in each genus must utilize different mechanisms for regulating chitinase-mediated tissue dissolution. Differing times for tissue dissolution may be necessary across these host species due to minor differences in host physiology, lifestyle, stage of infection, and viral activity.

An additional category of potentially important genes for baculoviruses concerning host-parasite interactions are host genes integrated and coopted by the parasite, such as the *chitinase* gene discussed previously. While in this survey we did not look specifically for such genes, we identified potentially horizontally acquired genes by using BLASTP to compare baculovirus proteomes to host proteomes in the NCBI database. Consistent with previous findings, we found eight sequences showing some similarity between host and viral sequences or to other sequences. We found *GTA*, *ubiquitin*, *IAP* (and *IAP-2*), *DNA ligase*, *chitinase*, *reductase 1*, *SNF2*, and UDP-glucosyltransferase genes, all of which were found previously (60). We also found two additional matches among hypothetical proteins of the gammabaculoviruses. While the first is an uncharacterized protease showing no signatures of positive selection (NLNVgp052), the second (found exclusively in Neodiprion lecontei nucleopolyhedrovirus [NeleNPV]) (NLNVgp006) shows high sequence similarity to the Drosophila Persephone gene. This gene plays a role in the activation of the Toll signaling pathway in response to fungi, with no known role in DNA virus recognition (perhaps due to a lack of a DNA virus model in Drosophila or other model systems) (65). Additionally, Persephone may be involved in immune response processes in Hymenoptera that are different from those in Drosophila, suggesting that it may have been coopted by NeleNPV.

Large double-stranded DNA viruses are expected to evolve differently from RNA viruses and smaller DNA viruses. With small genomes and no recombination, RNA viruses follow quasi-species models, in which high mutation rates result in a broad pool of genotypes all close to the fitness optimum. From these, a subset can be positively selected upon a change of environment (66). RNA viruses also have no true recombination (though they do have various levels of effective recombination and reassortment [67]), which can lead to the fixation of deleterious mutations in the genome, further facilitated by their high mutation rate (Muller's ratchet) (68). This can slow down the fixation of adaptive mutations in RNA viruses due to linked deleterious mutations (Hill-Robertson interference [69]). This may not hold true for large DNA viruses, such as baculoviruses, with large genomes and recombination allowing for the phasing of adaptive mutations in the same background (19, 70). We found that a large portion of the genome is under directional selection, either positive or negative, with a few key genes undergoing selective sweeps (Fig. 5 and 6). Unlike the model presented for the evolution of a quasi-species, which exists under mutation-selection equilibrium until a change to the equilibrium (66), the recombination facilitating replication in baculoviruses, as well as their various methods for escaping host suppression, results in the more complex evolutionary dynamics seen here. In contrast to observations for RNA viruses, we observed several separate selective sweeps as opposed to generally recurrent genome-wide sweeps. These aspects suggest that natural selection, both positive and background, should be more efficient, allowing for DNA viruses to fix beneficial mutations and purge deleterious mutations, with less hitchhiking of linked mutations. *dN*/*dS*, the direction of selection, and Tajima's D can be largely different between neighboring genes due to this recombination (Fig. 7).

Baculoviruses appear to adapt to more effectively infect their hosts through a combination of acquisition and cooption of host genes, accompanied by strong positive selection pressure on key replication genes. As some baculoviruses and nudiviruses are used as agents of biocontrol (24, 25), these viruses may be utilized more effectively by introducing a population of the virus with significant variation in genes that are commonly involved in antagonistic coevolution with the host, such as *chitinase*, the *LEF-4* to *LEF-5* loci, or *IAP-2*. Such variation might slow host adaptation to biocontrol methods, since the hosts would need to become resistant to multiple allelic variants. Overall, baculoviruses require the action of a diverse range of both virus and host genes to complete their life cycle, resulting in diverse signatures of adaptation. With increasing knowledge of baculoviruses and an improved understanding of their replication cycle, more targeted identification of factors key to replication and proliferation will leverage their more effective use in biocontrol and other activities.

## MATERIALS AND METHODS

### Data used in this study.

In our divergence-based analyses, we used previously assembled reference genomes from sequenced cloned viral particles, which provided fully phased natural haplotypes. We also used available annotations taken from the NCBI genome repository (https://www.ncbi.nlm.nih.gov/genome). These genomes are all for nudiviruses and baculoviruses, and their descriptions and accession numbers are given in Table S1 in the supplemental material. We excluded two genomes (Plodia interpunctella granulovirus [BVA96]) and Cydia pomonella granulovirus [CpGV]) because their sequences were close to identical to those of two other genomes used. We also excluded 7 other baculovirus genomes due to the difficulty in identifying orthology groups for genes, resulting in a data set of 64 viral genomes (Table S1). We also used a hytrosavirus, Musca domestica salivary gland hypertrophy virus (MdSGHV), as an outgroup for assembling the viral phylogeny.

Our polymorphism-based analyses used previously published data for Autographa californica multiple nucleopolyhedrovirus (AcMNPV) (43), sequenced using an Illumina Genome Analyzer IIx. We used 20 samples sequenced from 20 wild Autographa californica larvae from the sample population, all infected with AcMNPV, with only the AcMNPV genome sequenced. Each sample therefore represents a subpool of the total population's viral content, as each one likely harbored multiple different viral haplotypes. Sample descriptions are given in Table 4. We analyzed the samples separately and as a total pool.

### Identification of orthologous and paralogous genes.

For our divergence-based analyses, we identified groups of orthologous genes between the baculovirus and nudivirus genomes. Following a previously described method (71), we extracted the nucleotide and amino acid sequences of each gene by using the genome annotation and used the Basic Local Alignment Search Tool (BLAST) to perform pairwise BLASTN, BLASTP, and tBlastx searches (38) to identify matches across all species between nucleotide sequence pairs, amino acid sequence pairs, and nucleotide-amino acid pairs (BLAST parameters were E values of <0.001 for all comparisons and default parameters otherwise). We generated a list of orthologous and paralogous genes and checked for similar annotations by eye and by checking multiple-sequence alignments by eye (using --auto MAFFT parameters) (72). With this method, we generated 249 conservative sets of orthologous or paralogous gene sets across viral isolates (Data S3).

Gene function annotations were assigned using the best-described genome. When no function was known, we labeled the gene set with the hypothetical protein category. We attempted to group gene sets which did not group by the BLAST searches but shared a functional annotation by alignment. However, if alignment was not possible, we considered the gene sets separately during model fitting in PAML. To assign each orthology group a broad functional category, we performed a literature search for papers functionally characterizing the gene annotation in baculoviruses and nudiviruses.

### Molecular evolution of baculovirus genes across the phylogeny.

Following the identification of orthologous gene sets, we performed a multiple-sequence alignment and generated a tree (PRANK parameters: −codon +F for DNA) (41). For amino acid sequences found in all species (Data S1 to S3), we also concatenated each alignment and built a phylogeny by using PhyML (PhyML parameters: −d aa −m LG −b 100) to generate a species phylogeny (73). To look for gene-species tree discordance, we compared the gene trees to the species trees for all 20 conserved genes, alongside all supposedly fast-evolving genes, and we found little to no discordance so did not account for this in further analyses (42).

Using the gene trees and alignments generated by PRANK as described above, we performed a PAML-based analysis of molecular evolution by using the site test of selection in PAML with models 0, 1, and 2 (the site-based models) and models 7 and 8 (the beta site models) (40). These approaches give estimates of the ratio of nonsynonymous changes (*dN*) to synonymous changes (*dS*), with each test implemented to detect selective pressures under different assumptions. We used a likelihood ratio test to compare a neutral null model (model 0 or 7) with an alternative model including slightly deleterious mutations (model 1) or positive selection (model 2 or 8). We performed these analyses on all 249 gene sets and found the values of *dN* and *dS* for each model, along with the best-fitting model for each gene among the nested model sets (model 0, 1, or 2 and model 7 or 8). For the 20 genes found in all genomes, we estimated the values of *dN*/*dS* twice, once including nudivirus orthologs and once exclusively using baculovirus orthologs.

### Identification of nucleotide polymorphisms within AcMNPV.

Short-read data from each individual Autographa californica larva were trimmed using Sickle (minimum length = 20, minimum quality = 20) and aligned to the AcMNPV reference genome by using BWA MEM (BWA MEM parameters: −M −t 5) (74–76). Previous work using these data found a lowest frequency of polymorphisms of 0.00025 (43); we attempted to confirm this by using Lofreq, software designed specifically for pools that calls SNPs and estimates their frequency (45). Based on the lowest SNP frequency of 0.001, we could assume that there were close to 1,000 different viral genomes in the total population. We used this estimate to call SNPs across the 20 samples by using GATK Haplotypecaller (parameters: −ploidy 100) (44). Following this, we generated custom bed files for each sample and merged all bed files by using BedTools to find the total polymorphism in AcMNPV across the population (77), giving us SNPs called from approximately 1,000 viral genomes. We confirmed these SNPs and frequencies by using Lofreq and Popoolation2 (snp-frequency-diff.pl minimum count = 10, minimum coverage = 50, and maximum coverage = 20,000) (45, 78); we compared SNPs and frequencies found in each case and found no different SNPs and minor differences in frequencies. We cross-referenced these called variants with those called previously (43) to make sure that no sequencing errors were characterized as low-frequency variants.

### Identification of signatures of selection in AcMNPV.

We performed a genome-wide scan of polymorphisms by using the generated bed files to find Watterson's theta, Tajima's pi, and Tajima's D across sliding windows and across genes, using a custom Biopython script adapted from a previous work (79). We performed the McDonald-Kreitman test to find the neutrality index for all genes shared with AcMNPV's closest relative, Bombyx mori nucleopolyhedrovirus, using VCF files generated using GATK, the gene alignments generated using PRANK, and a custom Biopython script. We plotted these values by using R with the smoothing function Loess (smoothing = 0.1).

A founder effect caused by the bottlenecking of initial infection may affect signatures of selection (36). To control for this, we performed the McDonald-Kreitman test and found Tajima's D again after removing substitutions fixed within a single individual but absent in at least half of all other samples. Following this, we performed a *t* test to assess if the original and the subset of the data set differed significantly from each other.

We also assessed the conservation of position across neighboring genes in these genomes by identifying each AcMNPV gene's orthologs in other genomes (based on the orthology groups found previously). We then found each gene's neighboring gene in AcMNPV and all other genomes. We then found a proportion of conservation for each gene, based on the number of other genomes whose copy of this gene had the same neighboring genes as the AcMNPV ortholog versus the total number of orthologs, giving a conservation proportion.

### Recombination and linkage disequilibrium across the AcMNPV genome.

As recombination has previously been reported for nucleopolyhedroviruses (26), we attempted to see if this plays a role in adaptation to host infection. To look for linkage disequilibrium caused by these possible signatures of selection, we generated a consensus sequence for each of the 20 sequenced infected individuals by using GATK FastaAlternateReferenceMaker (44); this allowed us to generate sequences containing only substitutions which are fixed or at a high frequency (over 50%) in each host individual and to look for recombination between these variants across the total population. We used DNAsp to identify possible sites of recombination and *R*^2^ across the genome, using these consensus sequences (80).

We compared linkage disequilibrium to Tajima's D across the genome to help further identify signatures of selection, using LDheatmap in R (81).

### *F_ST_* values across infected individuals.

Taking the virus infecting each individual host as a separate population, we calculated *F_ST_* site by site across the genome pairwise between the viruses in all infected individuals, using Popoolation2 (parameters: suppress noninformative data, minimum count = 10, minimum coverage = 50, maximum coverage = 5,000, minimum covered fraction = 1, window size = 1, step size = 1, and pool size = 1,000) (78).

### Statistical software.

All statistical work was completed using R and the R packages LDheatmap and ggplot2 (81–83).

### Accession number(s).

The AcMNPV sequences from this study were submitted to GenBank under the accession numbers given in Table 4.

## Supplementary Material

Supplemental material
