# Supplementary material for: Baculovirus Molecular Evolution via Gene Turnover and Recurrent Positive Selection of Key Genes
Source: J Virol. 2017 Oct 27;91(22):e01319-17. doi: 10.1128/JVI.01319-17 (PMC5660496; doi:10.1128/JVI.01319-17)
Supplement: Supplemental material [file supp_91_22_e01319-17__index.html]

Baculovirus Molecular Evolution via Gene Turnover and Recurrent Positive Selection of Key Genes — Supplemental material 

# Baculovirus Molecular Evolution via Gene Turnover and Recurrent Positive Selection of Key Genes

## Supplemental material

- Supplemental file 1 -

  Table S1 (Viral genomes used in the divergence-based analysis.)

  Table S2 (Permutation test results for dN/dS for functional categories.)

  Data S1 (Orthology groups, their functional category, and the ORF IDs that are part of this functional group.)

  Data S2 (codeML summary statistics.)

  Data S3 (Codons under positive selection across ortholog groups.)

  Data S4 (McDonald-Kreitman test in AcMNPV.)

  Data S5 (Population genetics statistics across the genome.)

  XLSX, 1.0M
- Supplemental file 2 -

  Legends for Data S1 to S4.

  PDF, 63K
