# Supplementary material for: Baculovirus Molecular Evolution via Gene Turnover and Recurrent Positive Selection of Key Genes
Source: J Virol. 2017 Oct 27;91(22):e01319-17. doi: 10.1128/JVI.01319-17 (PMC5660496; doi:10.1128/JVI.01319-17)
Supplement: Supplemental material [file JVI.01319-17_zjv999183019s2.pdf]

Data S1: Orthology groups, their functional category and the ORF IDs that are part of this functional group.Data S2: codeML summary statistics. Each orthology group alongside their functional category, the number of genomes this group is found in, the best fitting models from codeML alongside the dN and dS values.

Data S3: Codons under positive selection across ortholog groups.

Data S4: McDonald-Kreitman test in AcMNPV. Table shows the gene, its position, the number of non-synonymous and synonymous substitutions and polymorphism in each gene, and the MK based results.

Data S5: Population genetics statistics across the genome.

Table 1: Summary of molecular evolution analyses grouped by functional category.

Functional Category	No. Genes	Sites Model (M0/M1a/M2a)				Beta Sites Model (M7/M8)			
		Pos.	Prop. Pos.	dN/dS >1	Median Percentile	Pos.	Prop. Pos.	dN/dS >1	Median Percentile
**Apoptosis**	6	0	0	2	0.715	2	0.333	1	0.684
**Capsid and Envelope**	20	4	0.2	3	0.539	6	0.286	2	0.644
**DNA Binding**	11	2	0.18	2	0.624	2	0.18	2	0.714
**Host Infection**	23	3	0.13	2	0.304	4	0.17	2	0.538
**Hyp. Protein**	158	21	0.13	29	0.571	17	0.107	30	0.464
**Replication**	14	5	0.35	4	0.524	5	0.35	4	0.670
**Transcription**	17	6	0.35	5	0.636	6	0.35	4	0.689
**All**	249	41	0.16	47		42	0.168	45	

Table 2: Generalized linear model for *dN/dS* versus functional group.

Variable	Estimate	Std. Error	t-value	p-value
**Intercept**	1.008	0.4571	2.205	0.0284
**DNA-binding**	-0.14755	0.07913	-1.865	0.0623
**Envelope and Capsid**	-0.6825	0.5221	-1.307	0.1924
**Host Infection**	-0.5455	0.5311	-1.027	0.3055
**Hypothetical Proteins**	0.1536	0.4671	0.329	0.7426
**Replication**	0.2681	0.5598	0.475	0.6353
**Transcription**	-0.2613	0.5431	-0.481	0.6309

Table 3: Logistic regression on the number of significantly selected codons site in each functional group.

Coefficients	Estimate	Std. Error	z-value	p-value
**Intercept**	-4.4236	0.1043	-42.406	<2e-16
**DNA-binding**	0.1730	0.1258	1.375	0.169
**Envelope and Capsid**	-1.2254	0.1400	-8.751	<2e-16
**Host Infection**	0.1870	0.1126	1.661	0.0467
**Hypothetical Proteins**	0.3512	0.1073	3.274	0.00106
**Replication**	-0.7675	0.1216	-6.310	2.80e-10
**Transcription**	0.8023	0.1351	0.821	0.244

Table 4: AcMNPV sequences used in the population level analysis.

Accession ID	Line ID	Read number
SRR1119903	S0	10019526
SRR1120009	S1	14944099
SRR1120065	S2	36195764
SRR1120362	S3	25939880
SRR1120103	S4	2806575
SRR1120106	S5	10893999
SRR1120170	S6	7927299
SRR1120269	S7	6695187
SRR1124059	S8	10479029
SRR1124050	S9	10214302
SRR1119904	T0	10181720
SRR1119940	T1	7814487
SRR1120007	T2	6921780
SRR1120010	T3	6863315
SRR1120012	T4	7481923
SRR1120015	T5	8247633
SRR1120016	T6	23589731
SRR1120102	T7	17090438
SRR1120104	T8	13058831
SRR1120105	T9	6349924

Table 5: Summary of the McDonald-Kreitman test within a population of AcMNPV.

Functional Category	Number of Genes	Neutrality Index (Direction of Selection)			
		**Mean**	**Median**	**Standard Deviation**	**FET *p*-value < 0.05**
**Apoptosis**	4	2.223	1.877	1.190	0
**Capsid**	6	1.051	0.870	0.435	0
**DNA Binding**	4	1.249	0.823	1.037	0
**Envelope**	12	1.310	1.048	1.019	0
**Host Infection**	16	1.334	0.861	1.086	1
**Hyp. Protein**	63	2.156	1.333	2.762	2
**Replication**	6	1.001	0.786	1.016	1
**Transcription**	14	1.509	0.833	1.616	0

Table 6: AcMNPV genes with strong signatures of positive selection within and between genomes.

Gene	Category	Position in AcMNPV (kb)	Mean dN/dS	MK DoS	Tajima's D	Positive selection across lineages	Positive selection in AcMNPV
Polyhedrin	Envelope	4.5	0.077	0.611	-5.463	N	Y
Env-Prot	Envelope	18.5	0.238	0.575	-4.644	N	Y
LEF-8	Transcription	40.5	0.269	0.430	-3.787	N	Y
LEF-3	DNA binding	57.7	0.308	2.793	-8.171	N	Y
IAP-2	Apoptosis	61	0.404	1.455	-4.684	N	Y
LEF-4	Transcription	76.5	1.264	0.762	-1.528	Y	Y
Helicase	Replication	80.6	2.092	0.223	-4.802	Y	Y
Chitinase	Host Infection	105.2	0.925	0.605	-1.907	Y	Y
V-CATH	Host Infection	106.9	0.251	0.671	-3.59	N	Y
p24	Capsid	109.9	0.229	1.182	-6.01	N	Y
e18	Envelope	125.1	0.161	3	-3.52	N	Y
ec27	Envelope	125.3	0.127	0.083	-3.615	N	Y
